# SPCA2 Regulates Orai1 Trafficking and Store Independent Ca^2+^ Entry in a Model of Lactation

**DOI:** 10.1371/journal.pone.0067348

**Published:** 2013-06-28

**Authors:** Brandie M. Cross, Anniesha Hack, Timothy A. Reinhardt, Rajini Rao

**Affiliations:** 1 Department of Physiology, Johns Hopkins University School of Medicine, Baltimore, Maryland, United States of America; 2 United States Department of Agriculture–Agricultural Research Service, National Animal Disease Center, Ames, Iowa, United States of America; Indiana University School of Medicine, United States of America

## Abstract

An unconventional interaction between SPCA2, an isoform of the Golgi secretory pathway Ca^2+^-ATPase, and the Ca^2+^ influx channel Orai1, has previously been shown to contribute to elevated Ca^2+^ influx in breast cancer derived cells. In order to investigate the physiological role of this interaction, we examined expression and localization of SPCA2 and Orai1 in mouse lactating mammary glands. We observed co-induction and co-immunoprecipitation of both proteins, and isoform-specific differences in the localization of SPCA1 and SPCA2. Three-dimensional cultures of normal mouse mammary epithelial cells were established using lactogenic hormones and basement membrane. The mammospheres displayed elevated Ca^2+^ influx by store independent mechanisms, consistent with upregulation of both SPCA2 and Orai1. Knockdown of either SPCA2 or Orai1 severely depleted Ca^2+^ influx and interfered with mammosphere differentiation. We show that SPCA2 is required for plasma membrane trafficking of Orai1 in mouse mammary epithelial cells and that this function can be replaced, at least in part, by a membrane-anchored C-terminal domain of SPCA2. These findings clearly show that SPCA2 and Orai1 function together to regulate Store-independent Ca^2+^ entry (SICE), which mediates the massive basolateral Ca^2+^ influx into mammary epithelia to support the large calcium transport requirements for milk secretion.

## Introduction

Secretory pathway Ca^2+^-ATPases (SPCA) are important in sequestering Ca^2+^ and Mn^2+^ from the cytoplasm into the Golgi and post-Golgi vesicles where they are important for post-translational modification, sorting and quality control of cargo proteins [1]. The two isoforms, SPCA1 (*ATP2C1*) and SPCA2 (*ATP2C2*) share significant sequence similarity, but have distinct distribution and function [2], [3]. SPCA1 is ubiquitously expressed in mammalian tissues where it serves an essential housekeeping function, as evidenced by embryonic lethality in the homozygous knockout mouse [4]. In contrast, expression of SPCA2 is confined to highly secretory or absorptive epithelia, including mammary, testis, salivary glands, intestinal tract and lung [2]. Because it shares similar transport characteristics as SPCA1, SPCA2 appears redundant at first glance. Recently, we showed pathologically elevated expression of SPCA2 in breast cancer derived tissue and cell culture models, leading to an investigation of its role in tumorigenicity [5].

We showed that SPCA2, but not SPCA1, traffics to the plasma membrane in the breast cancer derived MCF7 cell line, where it interacts with the store-operated Ca^2+^ channel Orai1 to elicit constitutive Ca^2+^ influx. This signaling function was independent of Golgi and ER Ca^2+^ stores, and did not require ATPase or transport function of SPCA2. Constitutive elevation of Ca^2+^ influx in these cells activated MAP kinase signaling pathways and promoted tumor proliferation. Knockdown of either SPCA2 or Orai1 expression was found to attenuate Ca^2+^-signaling and inhibit tumor growth in mouse xenografts. Analysis of mutants and chimeras revealed that the membrane-anchored C-terminal tail of SPCA2 was sufficient for interaction with Orai1 to elicit Ca^2+^ influx [5]. Interestingly, an alternative splice variant of *ATP2C2*, encoding a ∼20 kDa membrane-anchored C-terminal domain is expressed in several secretory tissues under control of the helix-loop-helix transcription factor MIST1 [6] suggesting a physiological role for Orai1-SPCA2 interaction. However, the functional significance of this interaction remains to be elucidated. Selective and prominent lactation-induced expression of SPCA2 in mammary epithelium [7], [8] provides a first insight into function.

Total calcium concentration in milk, including ionized Ca^2+^ and bound calcium, ranges between 40–80 mM in various mammalian species [9]. Multiple Ca^2+^ transporters, regulators and binding proteins must be upregulated to drive transcytosis of calcium while maintaining submicromolar cytoplasmic Ca^2+^ concentrations to avoid Ca^2+^ mediated toxicity and cell death. Transport and sequestration of Ca^2+^ is achieved by coordinated increase in the expression of Ca^2+^ pumps, and channels [10]. Current models suggest that polarized mammary secretory cells take up Ca^2+^ at the basolateral membrane via yet to be described Ca^2+^ channel(s) [9]. Ca^2+^ entering the mammary secretory cells then travels one of 2 routes to secretion into milk. In the first transport route (accounting for ∼40% of calcium in milk) Ca^2+^ is likely rapidly pumped in Golgi/secretory stores via SPCA1 and/or SPCA2 where it is bound to casein to facilitate casein micelle formation, packaged in secretory vesicles and secreted into milk primarily as Ca^2+^-casein [7], [8], [11]. The second transcellular route (accounting for ∼60% of calcium in milk) cell Ca^2+^ is pumped directly across the mammary secretory cells apical membrane into milk by PMCA2bw [12], [13]. The proteins involved in sequestering cell Ca^2+^ while it is shuttled to PMCA2bw are unknown but calbindin-9k is a candidate calcium binding protein that could provide this function [14]. An alternate but yet unproven role for PMCA2bw is that PMCA2bw may also pump Ca^2+^ directly into the secretory vesicles while it is trafficked to the apical membrane [12].

The least understood part of mammary calcium transport into milk is the mechanism by which calcium enters the basolateral membrane of the lactating mammary secretory cell. Based on our recent findings in mammary tumor cells, we evaluated a potential role for SPCA2 in eliciting Ca^2+^ entry into the lactating mammary secretory cells, by interaction with Orai1 channels [5]. In this study, we examine the interaction of SPCA2 with Orai1 channels throughout lactation, both in native mouse tissue and in a three-dimensional cell culture model derived from mammary epithelium (“mammospheres”). We show that SPCA2 and Orai1 are simultaneously induced early in lactation, colocalize, and are required for Ca^2+^ influx into mammary epithelial cells Our observations point to a new role for store-independent Ca^2+^ influx (SICE) in the sequestration of Ca^2+^ from the blood for transport to milk.

## Results

### Coordinated Induction of a Calcium Transporting Module in Lactation

**Figure 1:**
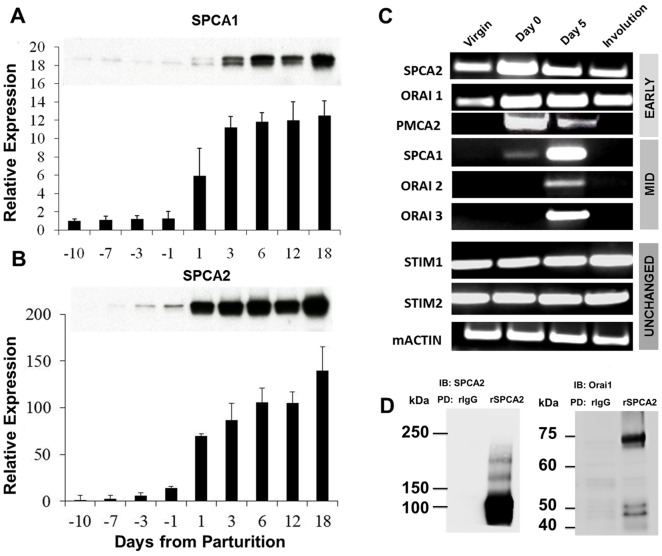
Expression profiles of calcium transporters in lactation. Mouse mammary tissue, starting from day 10 prior to parturition, was evaluated for the expression of (A) SPCA1, and (B) SPCA2 by Western blotting. Loading was normalized relative to tissue DNA concentrations, and expressed relative to starting levels at Day -10. C) RT-PCR of mRNA for isoforms of SPCA, Orai, PMCA and STIM proteins at distinct time points of mammary development, including pre-pregnancy (virgin), parturition (Day 0), lactation (Day 5) and involution (Day 5 after removal of pups). Transcripts are grouped as early, mid and unchanged, according to their time of induction following lactation on Day 0. D) SPCA2 was immunoprecipitated from lactating mouse mammary tissue using polyclonal rabbit anti-hSPCA2 peptide antibody or rabbit IgG as control. Orai1 was detected as a co-immunoprecipitate by immunoblotting (IB).

Lactation is characterized by massive transcellular flux of calcium, from the basolateral side of mammary alveolar epithelium (blood) into lumen (milk). This involves coordinated induction of a host of Ca^2+^ channels, transporters, buffering proteins and regulators, and must be tightly modulated to avoid cytoplasmic calcium toxicity. Previously, analysis of transcripts from mouse mammary gland tissue starting at Day 10 before parturition revealed induction of all three classes of Ca^2+^-ATPases, including isoforms of SERCA, PMCA and SPCA pumps [18]. Of note, while PMCA2b isoform showed the largest transcriptional induction of ∼100-fold, the secretory pathway Ca^2+^-ATPase was induced early and significantly prior to parturition. Subsequently, isoform-specific differences in mRNA were noted, with SPCA2 showing higher transcriptional elevation relative to SPCA1 in lactating mouse mammary gland [7], [8]. Here, we validate and quantify isoform-specific differences in SPCA proteins by Western analysis of mouse lactating mammary tissue. While the housekeeping isoform SPCA1 shows a substantive increase of ∼10-fold upon parturition (Figure 1A), SPCA2 protein is elevated by more than 100-fold (Figure 1B). Both ATPases remain elevated through lactation. Next, we evaluated transcriptional induction of Orai1 [19], an SPCA2-activated Ca^2+^ channel previously described to elicit Ca^2+^ influx in breast derived tumor cells [5]. Semi-quantitative PCR analysis showed early induction of Orai1, matching that of both SPCA2 and PMCA2 (Figure 1C). Two other isoforms, Orai2 and Orai3 were also transcriptionally induced, but at a later stage concordant with SPCA1 induction. STIM1 and STIM2, the calcium sensors in the ER, showed little relative change through the lactation cycle (Figure 1C). Previously, we had shown that SPCA2 interacts with Orai1 by co-immunoprecipitation from breast cancer derived MCF-7 cells and in HEK293 cells expressing a variety of chimeric and tagged proteins [5]. Here, we extended these observations to endogenous proteins expressed in native mammary tissue where such interactions may be physiologically relevant to lactation. Immunoprecipitation of SPCA2 from lactating (Day 5) mouse mammary tissue confirmed a physical association with Orai1 (Figure 1D).

### Distinct Localization of Two SPCA Isoforms in Lactating Mouse Mammary Gland

**Figure 2:**
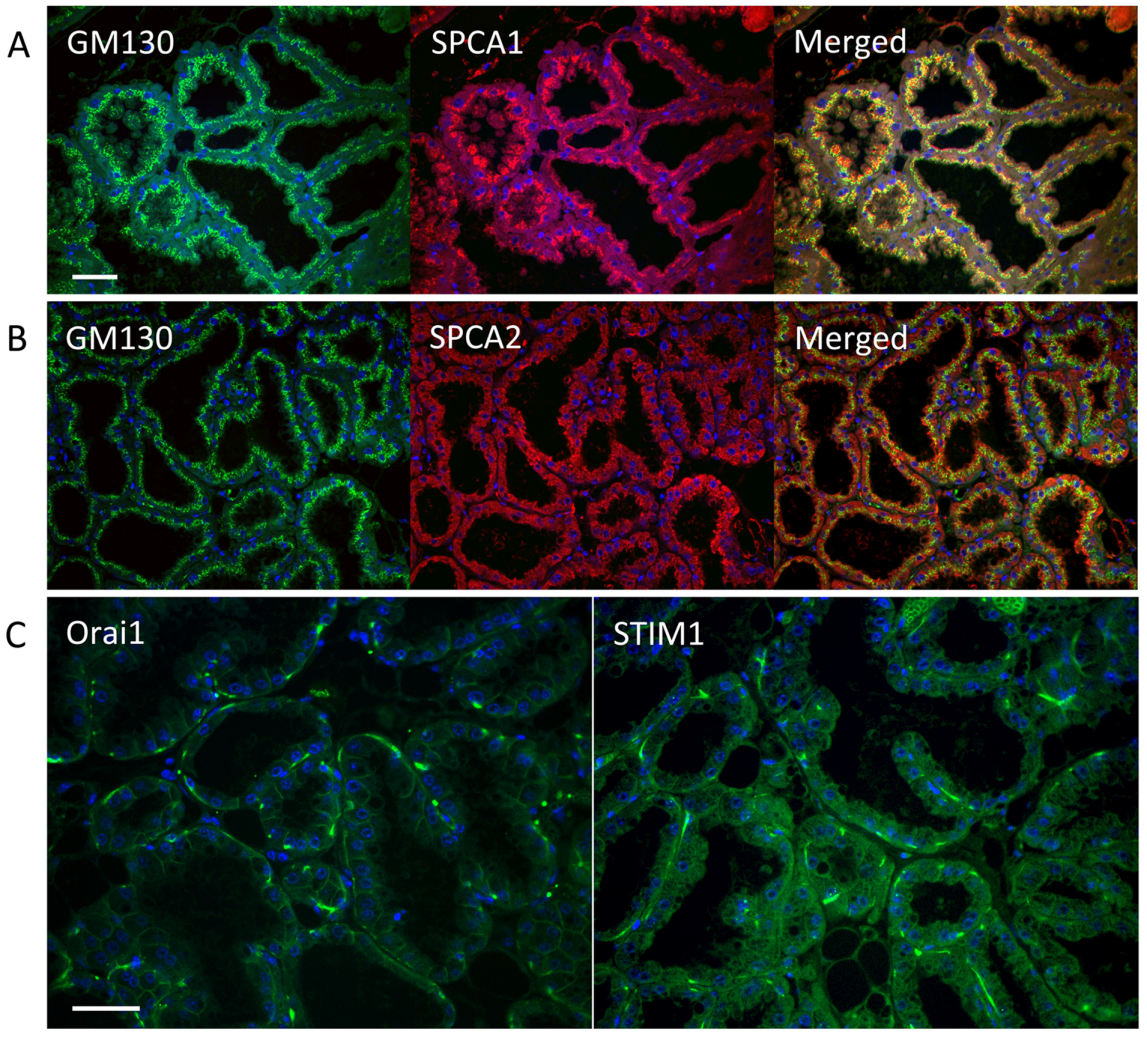
Immunofluorescence microscopy of SPCA1, SPCA2, Orai1 and STIM1 in lactating mouse tissue. Confocal microscopy imaging of sections treated as described under Experimental procedures. A) SPCA1 co-localizes with the Golgi marker, GM130. B) SPCA2 has a diffuse distribution, with little co-localization with Golgi marker, GM130. C) Orai1 (left panel) and STIM1 (right panel) show basolateral and reticular localization, respectively. Scale bar: 100 µm.

Previously, Faddy et al. [7] showed that whereas SPCA1 could be stained in all mammary gland cell types, including stromal and myoepithelial cells, expression of SPCA2 was restricted to the luminal epithelium, with no detectable staining outside the acini. Here, we show a further distinction between the two isoforms in subcellular localization as seen in Figure 2. Immunostaining of SPCA1 was discretely localized to compartments apical to the nucleus (Figure 2A), overlapping largely with the Golgi marker GM130 (Figure 2A; merge). In contrast, SPCA2 displayed a broader, punctate distribution with little colocalization with GM130 (Figure 2B). Thus, we conclude that in lactating mammary tissue SPCA1 has a conventional Golgi distribution, but SPCA2 is largely found in extra-Golgi vesicles. Secondary antibody controls and pre-block with antigenic peptides are shown in Figure S1. We also examined the distribution of Orai1 and STIM1 proteins. As expected for its known ER localization, STIM1 had a diffuse reticular distribution, whereas Orai1 was restricted to basolateral domains of the plasma membrane (Figure 2C). Both Orai1 and STIM1 stained myoepithelial cells, seen as patches in the tissue section. We conclude that SPCA2 and Orai1 are co-expressed in luminal epithelial cells of lactating mammary glands where their interaction may be important in mediating transepithelial Ca^2+^ flux.

### Expression and Localization of SPCA2 and Orai1 in Mammospheres

**Figure 3:**
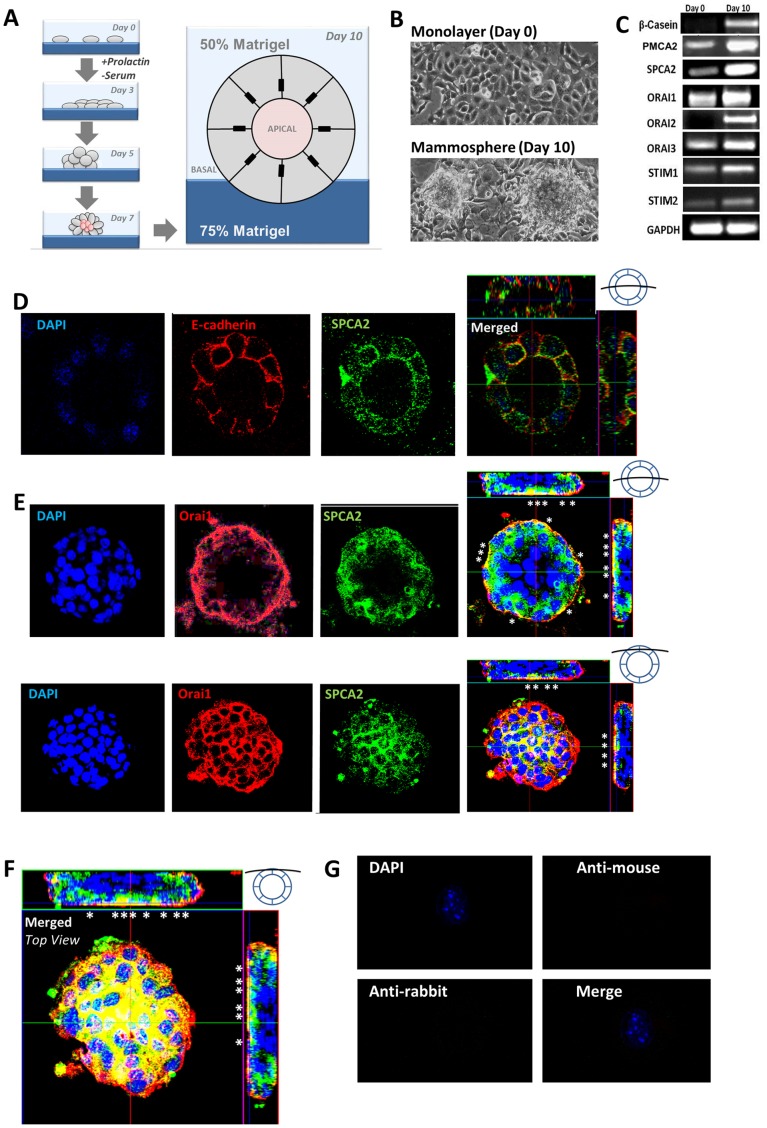
Expression of SPCA2 and Orai1 in mammospheres. A) Schema for induction of SCp2 cells into mammospheres. B) Cells in monolayer migrate and assemble into mammospheres following induction. C) Semi-quantitative RT-PCR showing that SPCA2, Orai1–3 and STIM1–2 are induced along with known markers β-casein and PMCA2 in the transition of SCp2 cell into differentiated mammospheres. D–F) Confocal sections of mammosphere showing indirect immunofluorescence of the indicated proteins; asterisks mark some areas of co-localization. Orthogonal sections are shown with the merged image. The cartoons indicate approximate location of optical plane through the mammosphere. D) Confocal section of a single layered mammosphere shows vesicular distribution of SPCA2 with some colocalization in the vicinity of E-cadherin as seen in the orthogonal view. E) Differentiated mammosphere showing Orai1 limited to the basal membrane with some SPCA2 punctae just below the plasma membrane, seen in orthogonal view. F) Top view of mammosphere shown in (E). Colocalized SPCA2 (green) and Orai1 (red) appear yellow. G) Secondary anti-mouse or anti-rabbit antibody alone shows no staining of mammosphere.

In order to evaluate potential functional roles in Ca^2+^ handling for SPCA2 and Orai1 in a lactation model, we turned to a three-dimensional culture that mimicked some aspects of native lactating tissue while still retaining the advantages of *in vitro* cell culture. The mouse mammary epithelial line SCp2 responds to basement membrane (Matrigel) and lactogenic hormone (prolactin) by differentiating into alveolus-like structures characterized by induction and secretion of milk protein, β-casein [15]. Formation of mammospheres with distinct lumen and tight junctions occurred over 10 days (Figure 3A–B). Transcriptional analysis revealed induction of β-casein in the mammospheres, confirming lactation-induced differentiation. We show increase of SPCA2, PMCA2 and Orai1 expression (Figure 3C), consistent with initiation of a lactogenic program for Ca^2+^ transport as seen in native tissue. Other Orai and STIM isoforms also showed varying levels of transcriptional induction (Figure 3C).

Immunofluorescence staining and confocal microscope imaging of mammospheres revealed punctate distribution of SPCA2 throughout the cell, reminiscent of mammary gland staining, with some concentration of puncta near the cell membranes. A merge with the basolateral marker E-cadherin showed apparent colocalization, although more careful evaluation of transverse sections suggests a juxtaposition of SPCA2 puncta immediately under the cell membrane (Figure 3D; Movie S1). Orai1 localization was enriched at the outer basal membrane of the mammosphere (Figure 3E) and a top view of the mammosphere showed a close juxtaposition of SPCA2 with Orai1 (Figure 3F; Movies S2 and S3). Secondary antibody controls showed no specific staining (Figure 3G). Taken together, these observations place a portion of SPCA2 at or near the basal membranes of mammospheres where it may be in position to functionally interact with Orai1 to regulate Ca^2+^ influx.

### SPCA2 and Orai1 are Critical for Ca^2+^ Entry in Mammary Epithelial Cells

**Figure 4:**
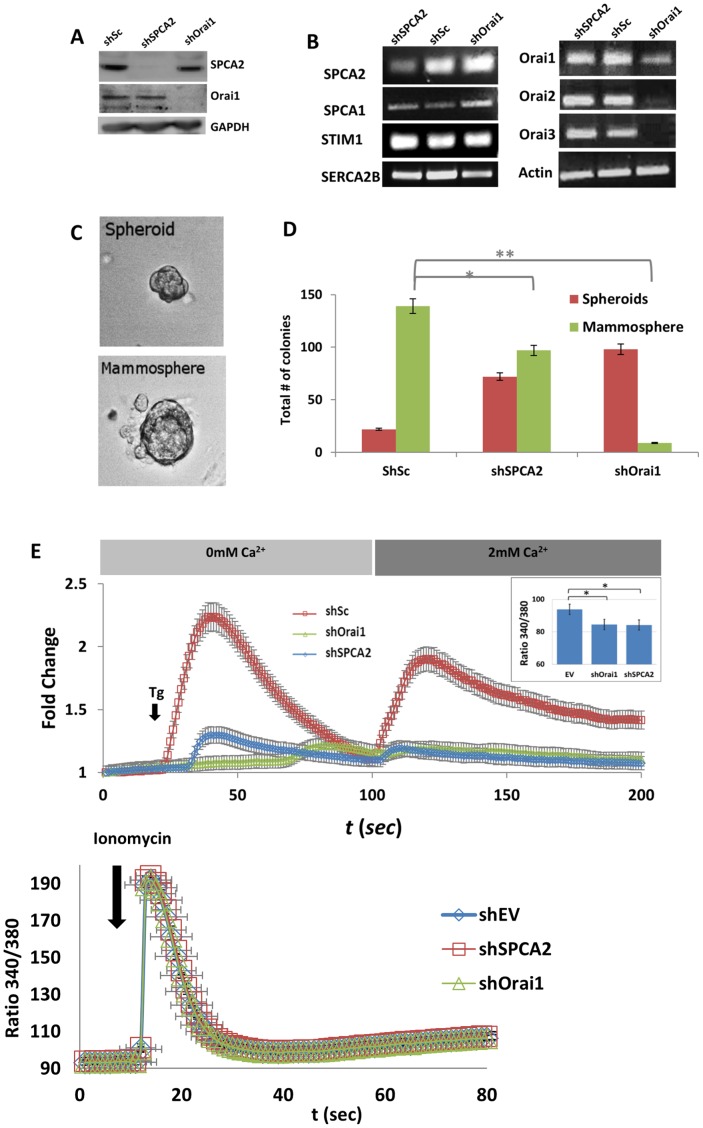
Effect of SPCA2 and Orai1 knockdown on mammosphere formation and Ca^2+^ influx. A) Western blot of SCp2 lysates derived from cells expressing shSc (scrambled shRNA), shSPCA2 and shOrai1 showing effectiveness of knockdown. B) RT-PCR of SPCA, Orai, SERCA2b and STIM1 isoforms in SCp2 cells following treatment with shRNA as indicated. Note that all three Orai isoforms were depleted in cells treated with shOrai1 virus. C) Comparison of small, aborted spheroids with mammospheres in SCp2 cells. D) Quantification of spheroids and mammosphere types in shRNA treated SCp2 cells ten days after lactogenic induction (* p≤0.05; ** p≤0.005). E) SOCE is drastically reduced in SCp2 monolayer cells treated with shSPCA2 and shOrai1 constructs (results averaged from n = 76 cells for shEV; n = 98 for shSPCA2; n = 123 for shOrai1). Fold change is the normalized change in fluorescence ratio (340/380 nm) of Fura-2. Inset: Fura2 fluorescence ratios showing baseline levels of free Ca^2+^ are lower in knockdown.

To investigate the potential contribution of SPCA2 and Orai1 in Ca^2+^ entry, we used shRNA constructs packaged in lentiviral vectors to knockdown their expression in SCp2 cells. Figure 4A is a Western analysis of cultured SCp2 cells showing significant reduction in expression of both proteins following transfection and selection of shRNA viral constructs. Examination of transcripts by semi-quantitative RT-PCR confirmed knockdown of SPCA2 and all three Orai isoforms (Figure 4B). We also noted small, potentially significant changes in transcript levels of SERCA2b (decreased) and SPCA1 (increased) in response to the knockdowns. SCp2 cells with either Orai or SPCA2 knockdown formed normal monolayers and grew at similar rates to control (scrambled shRNA), as seen in Figure S2A–B. Although Orai knockdown cells were able to polarize and form tight junctions as seen by the staining with E-cadherin (Figure S3A), mammosphere production was nearly absent, and was also noticeably decreased in shSPCA2 treated cells, with concomitant increase in number of small clumps of cells (spheroids; Figure 4C-D).

We examined the effect of SPCA2 and Orai1 knockdown on Ca^2+^ signaling pathways in monolayer SCp2 cells. Resting Ca^2+^ levels were significantly lowered in both SPCA2 and Orai1 knockdown cells (Figure 4E inset), consistent with our previous observation in HEK293 and tumor-derived MCF7 cells [5]. In control SCp2 cells, addition of thapsigargin blocks the SERCA2 Ca^2+^-ATPase resulting in passive release of SERCA2-filled stores, followed by store-operated Ca^2+^ entry (SOCE) upon reintroduction of extracellular Ca^2+^(Figure 4E). However, thapsigargin-induced Ca^2+^ release and subsequent Ca^2+^ entry were both largely diminished upon SPCA2 and Orai1 knockdown (Figure 4E). One interpretation was that ER stores and SOCE were both severely depleted in these knockdowns, however their normal growth and appearance was not consistent with ER stress or subsequent cell death (Figure S2). Indeed, total stored Ca^2+^ released by ionomycin was similar in control and knockdown cells (Figure 4F). Alternatively, a decrease in thapsigargin-releasable Ca^2+^ can be explained by a shift to thapsigargin-insensitive stores consistent with the transcriptional changes shown in Figure 4B. Previously, we showed that overexpression of the thapsigargin resistant SPCA1 pump in HEK293 cells blocked release of stored Ca^2+^ by thapsigargin and STIM1-mediated SOCE in response to thapsigargin [5]. These findings provide functional evidence for a major role in Ca^2+^ handling for SPCA pumps and Orai channels in a mammary epithelium.

### SPCA2 is Required for Cell Surface Trafficking of Orai1 in SCp2 Cells

**Figure 5:**
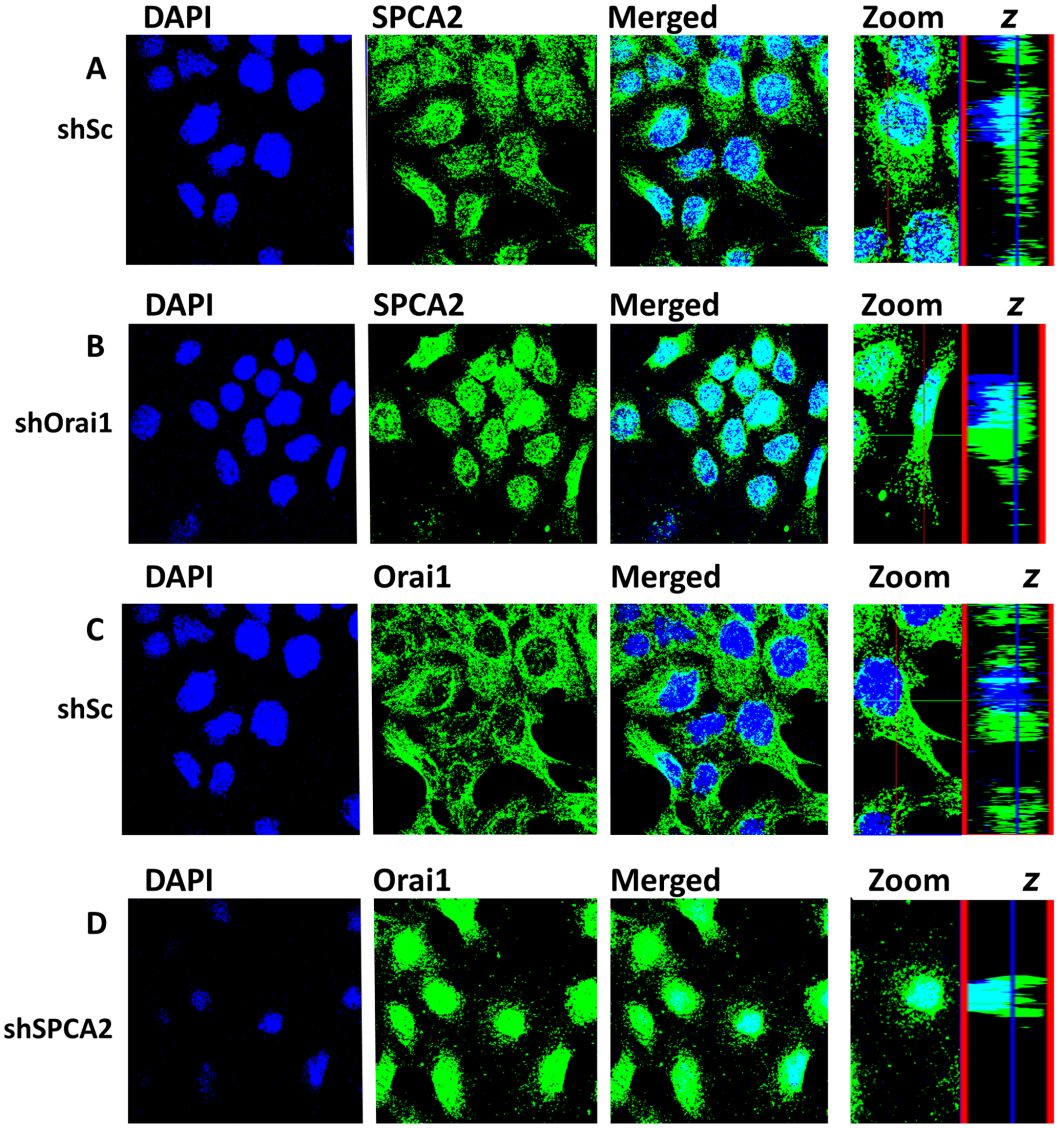
Depletion of SPCA2 blocks cell surface trafficking of Orai1. A–B) Immunofluorescence labeling of mSPCA2 shows less vesicular distribution of SPCA2 in cells treated with shOrai1. C-D) Immunofluorescence labeling of mOrai1 in SPCA2 knockdown cells shows retention to the perinuclear region only. SPCA2 and Orai1 signals from conjugated anti-rabbit and anti-mouse (AlexaFluor 288 and 388, respectively) secondary antibody were pseudocolored for ease of comparison.

Given the interaction between SPCA2 and Orai1, we considered the effect of gene knockdowns on their biogenesis and trafficking in mammary epithelial cells. In control SCp2 cells grown as monolayer, SPCA2 had punctate, perinuclear distribution whereas Orai1 showed both intracellular and plasma membrane staining (Figure 5A, C). There was minor colocalization of the two at the plasma membrane, as detected by cell surface biotinylation (not shown). Upon knockdown of Orai1, SPCA2 localization appeared more restricted to the perinuclear region, with less punctae near the plasma membrane (Figure 5B) although tight junction formation monitored by E-cadherin labeling appeared normal (Figure S3A). Strikingly, distribution of Orai1 largely shifted to the same perinuclear localization upon SPCA2 knockdown (Figure 5D). Consecutive confocal sections through individual cells showed that Orai1 localized around, but not in the nucleus in shSPCA2 knockdown cells (Figure S3B–D). Given that expression of the major Golgi Ca^2+^-ATPase SPCA1 was retained (Figure 4B), the results suggest that specific interaction with SPCA2 may be important for cell surface trafficking of Orai1 in mammary epithelium cells.

### C-terminal Domain of SPCA2 Partially Rescues Orai1 Trafficking and Restores Ca^2+^ Influx

**Figure 6:**
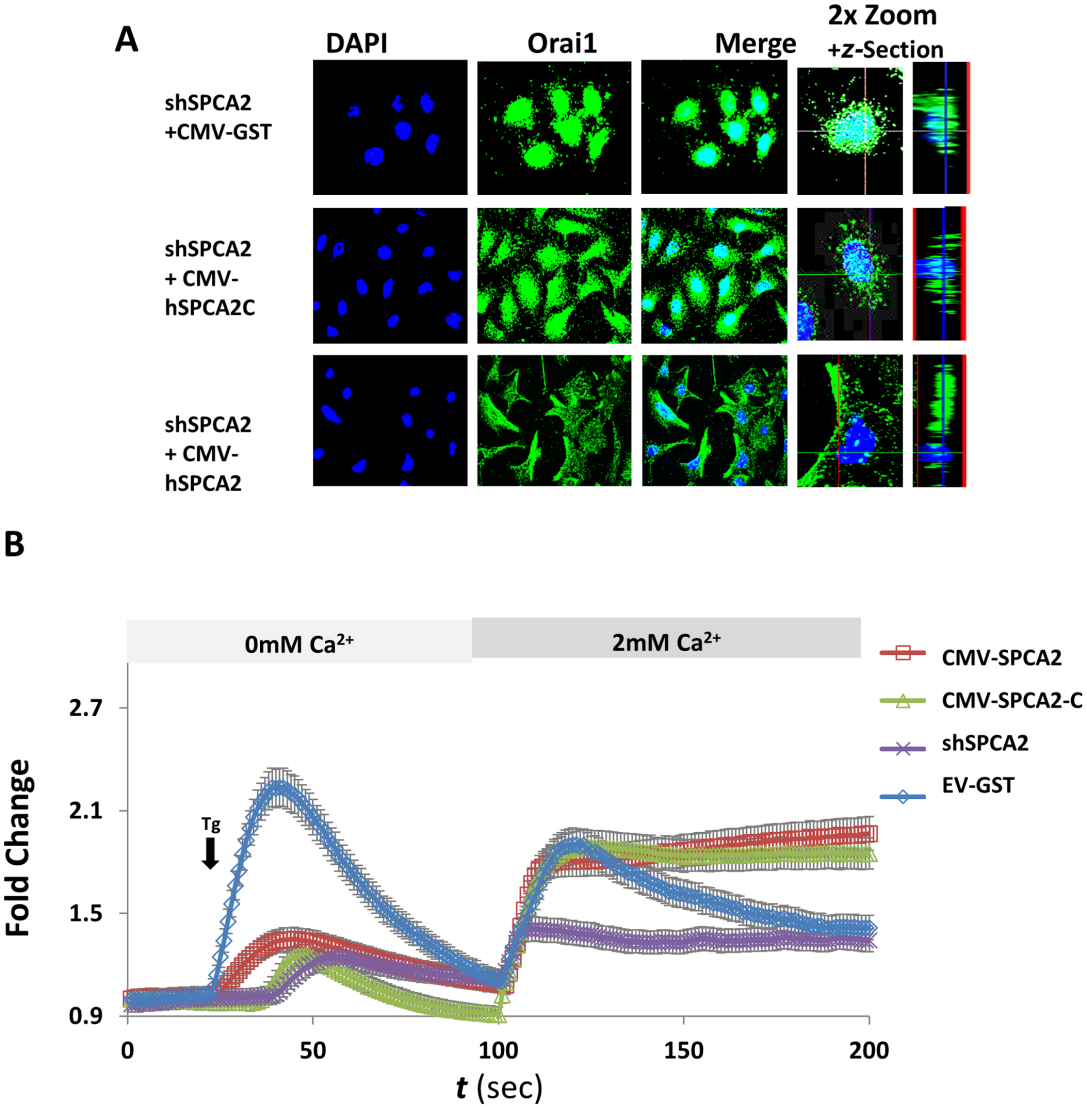
Ectopic expression of hSPCA2 constructs restores Ca^2+^ influx and Orai1 trafficking. A) Immunofluorescence staining of mOrai1 shows trafficking defect in shSPCA2 cells (top panel). When the C-terminal end of hSPCA2, hSPCA2-C is ectopically expressed mOrai1 staining appears more vesicular (middle panel). Full-length hSPCA2 fully rescues mOrai1 trafficking, showing both plasma membrane and vesicular localization seen (bottom panel). B) SOCE is restored for shSPCA2 treated cells ectopically expressing either full-length hSPCA2 or C-terminal domain, hSPCA2C (results averaged from n = 111 cells for EV/GST; n = 63 for shSPCA2/CMV-EV; n = 67 for shSPCA2/CMV-hSPCA2C and n = 75 for shSPCA2/CMV-shSPCA2). Fold change is the normalized change in fluorescence ratio (340/380 nm) of Fura-2. Error bars reflect standard deviation from the mean for each measurement and time point using a Student’s *t* test.

To confirm the role of SPCA2 in cell surface localization of Orai1 in mouse SCp2 cells, we reintroduced full-length, GST-tagged human SPCA2 into shSPCA2 treated cells using virally packaged vectors for efficient transfection. We also introduced the membrane-anchored C-terminal domain of hSPCA2 (hSPCA2C) previously shown to be necessary and sufficient to interact with Orai1 and elicit Ca^2+^ influx [5]. hSPCA2C was at least partially effective in redistributing Orai1 out of the perinuclear region to a more punctate location (Figure 6A). Full-length hSPCA2 fully reversed the effect of the knockdown, resulting in plasma membrane trafficking of Orai1 (Figure 6A).

shSPCA2 knockdown and re-transfected SCp2 cells were loaded with Fura-2 for Ca^2+^ imaging. Neither full-length or C-terminal domain of SPCA2 fully restored thapsigargin-sensitive stores (Figure 6B), again consistent with a shift to thapsigargin-insensitive stores. Strikingly, upon addition of extracellular Ca^2+^, Ca^2+^ influx was elevated and sustained with both constructs, to levels even higher than control (empty vector, EV-GST).

### Store-independent Calcium Entry (SICE) Requires SPCA2 and Orai1, and is Elevated in Mammospheres

**Figure 7:**
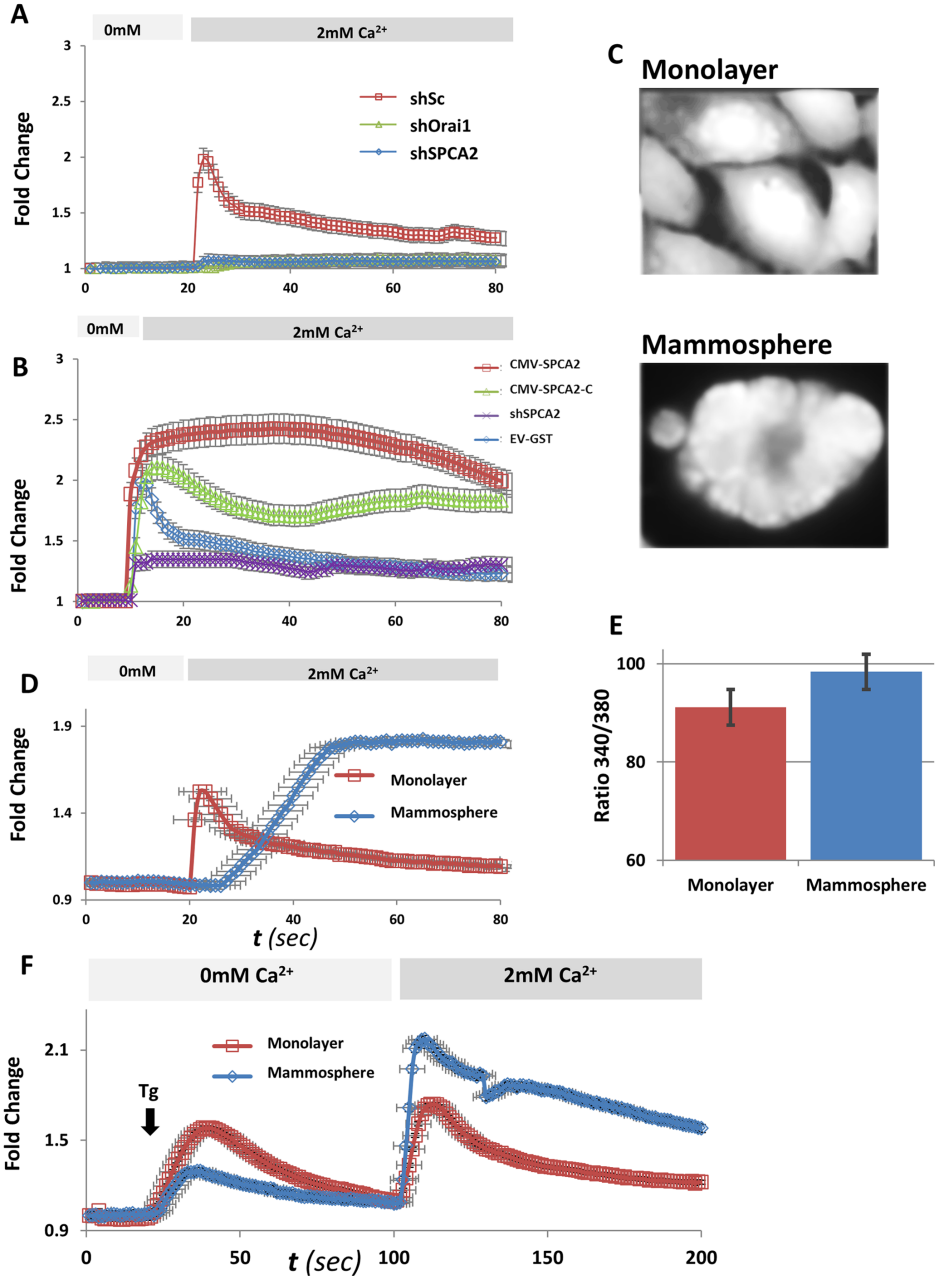
Store Independent Ca^2+^ Entry requires SPCA2 and Orai1. A) SICE is virtually abolished in monolayer cells expressing shRNA constructs for SPCA2 and Orai1 constructs (results averaged from n = 91cells for shSc; n = 82 for shSPCA2; n = 92 for shOrai1). Error bars reflect standard deviation from the mean for each measurement at each time point. B) SICE is restored and elevated in shSPCA2 treated cells expressing either hSPCA2 or hSPCA2C (results averaged from n = 94 cells for EV/GST; n = 74 for shSPCA2/CMV-EV; n = 91 for shSPCA2/CMV-hSPCA2C and n = 104 for shSPCA2/CMV-shSPCA2). C) Fluorescence image of Fura-2AM loaded in monolayer cells, and mammospheres. (D) Store independent Ca^2+^ influx in monolayer cells and differentiated mammospheres. After a brief (∼20 s) incubation in nominally Ca^2+^ free extracellular medium, readdition of Ca^2+^ (2 mM) elicits Ca^2+^ entry that is transient in monolayer cells but elevated and sustained in mammospheres (n = 129 and n = 84 for monolayer and mammosphere cells, also respectively). E) Basal Ca^2+^ levels are similar in mammospheres, relative to monolayer cells as seen by Fura-2 fluorescence ratio. F) Store-dependent Ca^2+^ influx in monolayer cells and differentiated mammospheres. Addition of thapsigargin (Tg) empties the internal (ER) stores in nominally Ca^2+^-free medium. Upon readdition of extracellular Ca^2+^ (2 mM), Ca^2+^ influx is slightly higher in mammospheres (n = 89 and n = 67 for monolayer and mammosphere cells, respectively). Fold change is the normalized change in fluorescence ratio (340/380 nm) of Fura-2.

Recently, we obtained evidence for a novel mode of store independent Ca^2+^ entry (SICE) elicited by SPCA2 via interaction with Orai1 (5). Following brief exposure of SCp2 monolayer cells to nominally Ca^2+^ free medium, reintroduction of extracellular Ca^2+^ was accompanied by rapid and transient influx that was abolished by knockdown of either SPCA2 or Orai channels (Figure 7A). Addition of thapsigargin to SCp2 at 5–30 second intervals following transfer to Ca^2+^ free medium demonstrated that the stores were largely unchanged (Figure S4A) as seen by the rate of Ca^2+^ release and peak height. Therefore, the Ca^2+^ influx observed in Figure 7A is not likely to be SOCE. Furthermore, we evaluated maximal SOCE in these cells by comparing thapsigargin-elicited elevation of Ca^2+^ levels in the presence or absence of extracellular Ca^2+^ (2 mM), shown in Figure S4B. Maximal SOCE estimated by this method was significantly smaller than that observed in Figure 7A. Therefore we conclude that SPCA2 and Orai1 contribute to a store-independent mechanism of Ca^2+^ entry (SICE) that is unmasked upon brief removal of extracellular Ca^2+^. As additional demonstration of this mechanism of Ca^2+^ entry, we evaluated SICE in shSPCA2 treated cells that were transfected with full-length or C-terminal domain of hSPCA2. Both constructs were able to confer elevated and sustained Ca^2+^ entry to the SPCA2 knockdown cells, consistent with plasma membrane delivery and functional rescue of Orai1 (Figure 7B). These results also suggest that a portion of the Ca^2+^ entry observed after thapsigargin addition, as seen in Figure 6B, was due to SICE.

Finally, we investigated whether this store-independent mode of Ca^2+^ entry occurred in mammospheres, where SPCA2 and Orai1 are induced upon differentiation. Monolayer and mammosphere cells (Figure 7C) were loaded with Fura2-AM, washed and imaged. In differentiated mammospheres, SICE occurred with slower kinetics relative to monolayer cells, and remained elevated and sustained (Figure 7D), slowly returning to baseline (not shown). Basal Ca^2+^ concentrations were similar in mammospheres and monolayer SCp2 cells (Figure 7E). It is possible that the slow rise in Ca^2+^ upon induction of SICE is due to slower recruitment of SPCA2-containing vesicles to the mammosphere basal membrane, relative to STIM1, as well as active Ca^2+^ sequestration consistent with transcriptional induction of SPCA pumps in mammospheres. Thapsigargin elicited a smaller release of Ca^2+^ in mammospheres (Figure 7F), again consistent with induction of thapsigargin-insensitive SPCA pumps, but Ca^2+^ entry was significantly larger. Taken together, we propose a model in which the induction and interaction of Orai1 and SPCA2 upon lactogenic differentiation results in increased store-independent Ca^2+^ influx.

## Discussion

### Orai1 Mediates Basolateral Ca^2+^ Influx in Mammary Epithelium

Polarized localization of Orai1 in secretory epithelium has previously been investigated only in pancreatic acinar and salivary gland cells. In pancreatic acinar cells, where secretagogue-induced Ca^2+^ signaling is accompanied by robust Ca^2+^ extrusion and refilling of stores, Orai1 co-localized with STIM1 in puncta along the basolateral membrane [20], consistent with a role in store-operated Ca^2+^ entry. Paradoxically, however, the bulk of Orai1 was seen in the apical membrane where it co-localized and interacted with IP3R but not STIM1. Thus, additional roles for Orai1 separate from SOCE, are likely and remain to be elucidated. An independent study by Hong et al. [21] localized Orai1 to both apical and lateral membranes of pancreatic acinar cells. These studies highlight the possibility of store-independent roles for this so-called “store-operated” Ca^2+^ influx channel.

The molecular identity of the calcium influx pathways in mammary epithelial cells has been a mystery. A genome-wide screen revealed several voltage-gated and TRP channels that were expressed but not increased dramatically during Day 1 of lactation [13]. McAndrew et al. [22] reported an increase in Orai1, but not Orai2 or Orai3 transcript in lactating mice. Here we show early induction of Orai1, concomitant with calcium pump isoforms SPCA2 and PMCA2, whereas Orai2–3 isoforms are elevated later in lactation (Day 5, post parturition), as seen for SPCA1. Thus, the Orai channels are good candidates for Ca^2+^ entry in mammary epithelial cells. This was substantiated by a distinct localization of endogenous Orai1 to the basolateral membrane of lactating mammary epithelia and in differentiated mammospheres cultured in vitro. Finally, knockdown of all three Orai channel isoforms abolished store-independent Ca^2+^ entry in SCp2 cells, implicating a prominent role for these channels in basolateral influx.

### SPCA2 Interacts with Orai1 to Mediate Store-independent Ca^2+^ Entry in Mammary Epithelium

Previously, we had demonstrated an unexpected moonlighting function of SPCA2, independent of pumping activity, in which the N- and C- termini interacted with Orai1 to elicit Ca^2+^ entry into cells. A consequence of this signaling activity was the oncogenic role of SPCA2 in breast cancer cells, where dysregulation of expression led to constitutive Ca^2+^ influx, activation of MAP kinase pathways, high rates of proliferation and tumorigenesis. Furthermore, SPCA2-mediated activation of Ca^2+^ entry appeared to be independent of both Golgi and ER stores, and of the STIM sensors [5]. Thus, in non-polarized cells, this unusual property would result in a futile cycle of energy independent activation of Ca^2+^ entry, via Orai1, and energy (ATP)-requiring Ca^2+^ efflux via active sequestration of Ca^2+^ into the secretory pathway. However, SPCA2 is restricted to polarized cells of secretory or absorptive epithelia (intestinal, lung, mammary and other glands; [2]). Therefore, we hypothesized that induction of SPCA2 expression in polarized cells may allow activation of Ca^2+^ influx channels at one membrane domain, and active transport of Ca^2+^ at the other membrane domain, to facilitate transepithelial transport of Ca^2+^ across the epithelium. Here, we provide evidence for a robust physiological role of SPCA2 in promoting SICE in a model of lactating mammary epithelia.

Although both SPCA isoforms are upregulated during lactation, we found that SPCA2 was elevated earlier and to significantly higher levels, compared to SPCA2. Whereas SPCA1 showed discrete localization to Golgi compartments, SPCA2 was predominantly localized to a vesicular compartment, closely associated with both apical and basolateral membranes. These differences are consistent with non-redundant roles for two isoforms of the secretory pathway pumps in mammary epithelium and suggested an isoform-specific critical function for SPCA2 in lactation. We began by showing co-expression, partial colocalization and co-immunopreciptation of endogenous SPCA2 with Orai1 in lactating mouse mammary epithelium and in differentiated mammospheres. Next, we showed significant induction of SICE concomitant with induction of Orai1 and SPCA2 upon lactogenic differentiation of SCp2 cells into mammospheres. Finally, knockdown of either SPCA2 or Orai channels virtually abolished SICE in mammospheres. Taken together, these data provide evidence for a physiologically relevant function for SPCA2 in the activation of Orai1. Further investigation should reveal whether SPCA2 also interacts with other Orai isoforms in lactation.

We note that a C-terminal transcript of SPCA2 is expressed under control of MIST1, a beta helix-loop-helix transcription factor, in pancreatic acinar cells [6]. This ∼20 kDa fragment lacks ATP and Ca^2+^ binding sites, and therefore, has no transport function. However, we found that a minimal membrane-anchored C-terminal domain is sufficient to activate Orai1 and elicit Ca^2+^ influx. It remains to be determined whether native expression of this membrane-embedded C-terminal domain also mediates SICE in various secretory epithelia by interaction with basolateral Orai1.

### Interaction between SPCA2 and Orai1 is Required for Orai1 Trafficking and SOCE in Mammary Epithelium

The severe reduction in thapsigargin-sensitive but not ionomycin-sensitive stores upon knockdown of Orai channels suggested a shift in expression and/or localization of thapsigargin-insensitive SPCA pumps. We show a redistribution of SPCA2 to a more perinuclear location, continuous with the nuclear membrane, in the absence of Orai channels. More dramatically, distribution of endogenous Orai1 was limited to a similar perinuclear reticular compartment in shSPCA2 cells, suggesting an arrest in trafficking out of the endoplasmic reticulum. Distinct from conventional endoplasmic reticulum, this specialized sub-compartment remains to be identified. The biogenesis defect was effectively reversed by introducing hSPCA2 into the knockdown cells, ruling out any non-specific effects of the shRNA reagent, with concomitant functional restoration of Ca^2+^ influx. Given the expression of SPCA1 in these cells, we reasoned that Orai1 trafficking did not require secretory pathway/Golgi Ca^2+^ stores. Indeed, the ability of the C-terminal domain of SPCA2 to partially mediate Orai1 exit from this perinuclear compartment suggests that Orai1 trafficking requires a chaperone-like interaction with SPCA2 protein. Further studies using longer fragments, or chimeric constructs with both N- and C-termini implicated in Orai1 binding [5], may narrow down the precise domains of SPCA2 that mediate this function. It also remains to be seen whether SPCA2 facilitates Orai1 trafficking in other secretory epithelia where it is expressed.

In conclusion, we demonstrate an isoform-specific physiological role for SPCA2 in lactating mammary epithelium. This role is mediated in large part by interaction with Orai1, although additional Ca^2+^ entry channels may also be involved. These studies lay a framework for the investigation of SPCA2 function in other secretory or absorptive epithelia, where high expression levels have been documented. Prior to this study nothing was known about the mechanisms that mediate basolateral calcium entry into the mammary secretory cell to support calcium needs for milk production. The data presented here demonstrate that SPCA2 and Orai1 function together to regulate SICE, which mediates the massive basolateral Ca^2+^ influx into mammary epithelia to support the large calcium transport requirements of lactation.

## Methods

### Animals

All animal work was conducted according to relevant national and international guidelines. The National Animal Disease Center’s Animal Care and Use Committee approved all animal procedures. Pregnant or lactating 129/SV mice were housed individually, in hanging basket cages on sawdust bedding. All mice were equalized to 6 pups per mouse mother on day one of lactation. Mice were killed at times indicated. Mice were anesthetized with a 50∶50 mix of CO_2_:O_2_ followed by decapitation. Mammary tissue was removed and fixed as described below or flash frozen in liquid N_2_, and stored at −70°C until processed as described.

### Cell Culture

SCp2 cells (gift of Andrew Ewald, Johns Hopkins University) [15] were cultured in monolayer media-DMEM/F-12 (1∶1) with 5% HI-FBS and insulin (5 µg/ml) at 37°C in a 5% CO_2_ incubator with ample humidity. Upon 80% confluence, they were trypsinized in 0.25% Trypsin with EDTA (Invitrogen) and centrifuged at 8 rpm for 2 minutes to remove excess trypsin. Cell growth was monitored using Cell Titer 96 AQ from Promega according to manufacturer’s instructions. For mammospheres, trypsinized cells were then mixed with a solution containing 50% Matrigel (Sigma) and 50% differentiation media (DMEM/F-12 (1∶1), hydrocortisone (1 µg/ml), insulin (5 µg/ml) and recombinant mouse prolactin (3 µg/ml). Cells were plated at 1×10^3^ on a 100% Matrigel matrix on 25 mm round coverslips. Matrix was again allowed to solidify prior to the addition of differentiation media to cover the layered coverslip. Mammosphere culture was allowed to differentiate for 10 days, changing the media every second day, prior to assays for differentiation.

### Mammosphere Culture

SCp2 cells were plated on 25 mm coverslips for three-dimensional culture at 10^3^ cells per coverslip. These coverslips were placed in a standard cell culture incubator and allowed to differentiate. Differentiation liquid media was changed every two days and replaced with fresh media. Cellular clusters (consisting of dense groups of >4 cells each) were counted and categorized into one of three categories: (1) undifferentiated clusters- cells which maintained characteristic of monolayer culture but were in close proximity to one another (single cells); (2) spheroids- partially differentiated cell clusters with characteristics which showed lack of tight junction formation and lumen; (3) mature mammospheres- cells which grouped into a differentiated mass with tight junctions and a lumen. All three classifications were considered clusters (belonging to group 1) but the proportion of spheroids to mature mammospheres and the percentage of cells, which went through the entire development program, were noted.

### RNA Collection and RT-PCR

10^6^ cells were washed with sterile Hank’s Balanced Salt Solution (HBBS; Invitrogen) prior to RNA extraction. An RNAeasy kit was used to collect the total RNA via centrifugation (Qiagen) per the manufacturer’s instructions and quantified by absorption at 260 nm by spectrometry. RNA was converted to cDNA by reverse transcription using iScript (Bio-Rad Laboratories). Equal amounts of cDNA was subjected to PCR amplification for specific transcripts using the following primers: Orai1∶5′-ACCCCACGAGCGCATGCATC-3′ (*forward*) and 5′-GCTTGGTGGGGCTTGGCTGT-3′ (*reverse*); Orai2∶5′-CTGAGGTGGTCCTGCTCT-3′ (*forward*) and 5′-GGTAGAAGTGGATGGTGAAG-3′ (*reverse*); Orai3∶5′-CATCCACAATCTCAACTCTG-3′ (*forward*) and 5′-ATAGAAGCAGAGGATGGTGT-3′ (*reverse*); STIM1∶5′-AAGAGTCTACCGAAGCAGAG-3′ (*forward*) and 5′-GTGCTATGTTTCACTGTTGG-3′ (*reverse*); STIM2∶5′-GTGCGCTGGGTCGGAAGAC-3′ (*forward*) and 5′-GGGGCACCAGATCGCATCG-3′ (*reverse*); SPCA1∶5′-CCAGTGTGGCCGTGGCTGAC-3′ (*forward*) and 5′-TCAGCCTGGAGAAGGCCTGCAA-3′ (*reverse*); SPCA2∶5′-GACCTGCTGCTGCTGACGGG-3′ (*forward*) 5′-CAGGCCAGAGGCACCCAAGC-3′ (*reverse*); PMCA2∶5′-CAGGGTCTGCCACCCTCGGAG-3′ (*forward*) and 5′-CATGGTCGGGACAGCTCCCCTA-3′ (*reverse*); SERCA2B: 5′-ACTTCTTGATCCTCTACGTG-3′ (*forward*) and 5′-AGACCAGAACATATCGCTAA-3′ (*reverse*); actin: 5′-GCAGCTCCTTCGTTGCCGGT-3′ (*forward*) and 5′-TACAGCCCGGGGAGCATCGT-3′ (*reverse*). The number of amplification cycles was adjusted to ensure that generation of products was not saturated. Loading control was mActin.

### Membrane Preparation and Western Blotting

Mammary tissue microsomes were prepared as previously described [12]. Briefly, tissue was homogenized in 10 volumes of Buffer A containing Tris–HCl (10 mM), MgCl_2_ (2 mM), sucrose (300 mM) and a complete protease inhibitor cocktail (Boehringer Mannheim Indianapolis, IN) at pH 7.5. The homogenate was mixed with an equal volume of Buffer B (Buffer A plus 0.3 M KCl) and centrifuged at 4000 g for 10 min. The supernatant was collected, adjusted to 0.7 M KCl, and centrifuged at 100,000 g for 1 h. The supernatant was discarded and the pellets were resuspended in Buffer C (Buffer A plus 0.15 M KCl).

Microsomes from mammary tissue were incubated for 15 min at room temperature in a modified Laemmli buffer containing 150 mg/ml urea and 65 mM DTT. Samples were then electrophoresed for 50 minutes at 200 volts in a 4–12% Novex NuPAGE® Bis-Tris Gel using MOPS SDS running buffer (Life Technologies, Grand Island, NY). Proteins were transferred to nitrocellulose membranes using the iBlot Dry Blotting System (Life Technologies, Grand Island, NY) at 23 volts for 7 minutes. The blots were blocked with StartingBlock T20 (Thermo Fisher Scientific Inc). SPCA1 (RS-1 #227) or SPCA2 (orange #2) antibodies were diluted 1/2000 in StartingBlock T20 and the blots were incubated over night at 4°C. After washing they were incubated 1 hr at RT with 1/50000 HRP goat anti-rabbit #31460 (Thermo Fisher Scientific Inc) and washed. Blots were developed using Pierce’s Supersignal (Pierce Products, Rockford IL) using the protocol provided by the manufacturer. Developed film was imaged and bands quantitated with a Gel Doc EZ imager (BioRad, Hercules, CA).

1×10^6^ cells from cell culture were washed three times with sterile HBBS (Invitrogen) then immediately lysed in buffer containing 20 mM Tris-HCl, 150 mM NaCl, 1 mM Na_3_EDTA, 1 mM EGTA, 5 mM Na_4_P_2_O_7_, 1 mM Na_3_VO_4_, 10 mM NaF, pH 7.4 with 1% SDS in the presence of protease inhibitor cocktail (Roche). Protein concentrations were assayed using a bicinchoninic acid assay kit (Pierce). Approximately 50 µg of protein was loaded onto a NuPAGE gel for SDS-PAGE Analysis and Western blotting. Immunoblots were made on 0.45 µm nitrocellulose membrane (Bio-Rad). Antibodies for mouse SPCA2, Orai1 and GAPDH were incubated with the membrane overnight at 1∶1000 dilution in 1×PBST (PBS plus 0.2% Tween). Membranes were washed five times in PBST then mouse or rabbit secondary antibody conjugate to HRP was added (GE Healthcare UK Limited) at 1∶2000 for one hour at room temperature on a rotator and the membranes were washed three times with PBST then four times with normal PBS to remove Tween residue. Blots were visualized using ECL kit (Pierce) on an Intelligent Dark Box Imager (Fuji Film).

### Immunofluorescence Microscopy

Mammary tissue was fixed in Tellyesniczky’s fixative (70% ethanol, formalin, glacial acetic acid, 20∶2∶1) for 5 h at room temperature [16] and then stored in 70% ethanol prior to paraffin embedding. Paraffin-embedded sections were cleared in xylene and rehydrated through an alcohol series. Tissue sections were immersed in antigen unmasking solution H-3300 (Vector Laboratories; Burlingame, CA) in a preheated pressure cooker. Sections remained in the pressure cooker for 20 minutes after the pressure maximized. The sections were then allowed to cool, wash 3 times with distilled, deionized water followed by PBS for 5 minutes. After permeabilization/blocking of the sections with PBS containing 0.5% Triton X-100,.01 g sodium azide and 50 mg/ml BSA, the primary antibodies were applied and incubated at 4°C overnight. Primary antibodies used were 1/50 Orai1 # 4281 (ProSci Incorporated, Poway, CA), 1/50 Stim1 # 610954 (BD Transduction Laboratories, San Jose, California), 1/100 GM130 #610822 (BD Transduction Laboratories, San Jose, California). SPCA1 (1/100) and SPCA2 (1/100) antibodies were prepared and characterized as previously described [11], [17]. The slides were washed 3 times in blocking buffer and then incubated for 2 hr at 37°C using secondary antibodies Green anti-mouse at 1∶1000 Alexa Fluor A11017 488 F(ab’)_2_ fragment of goat anti-mouse IgG(H+L), and Red anti-rabbit at 1∶1000 Alexa Fluor A11070 594 F(ab’)_2_ fragment of goat anti-rabbit IgG (H+L) (Molecular Probes/Life Technologies, Grand Island, NY). The slides were washed 3 times with blocking buffer and mounted with VectaShield (Vector Laboratories). Slides were viewed and photographed on Zeiss Axio Scope.A1 fluorescence microscope (Carl Zeiss, Germany).

Monolayer cultures were washed three times with 1×PBS prior to fixation in 4% paraformaldehyde and subsequent permeation in blocking buffer (1% BSA and 0.5% Triton-X in 1xPBS). Cells were then blocked and permeabilized in 0.1% Triton and 1% BSA in 1xPBS and washed three times in 1xPBS. Cells were incubated with antibodies to SPCA2 [5], Orai1 (Sigma) and E-cadherin overnight at 4°C. The next day cells were washed again (three times in 1xPBS) and incubated with conjugated anti-rabbit and anti-mouse (AlexaFluor 288 and 388, respectively) for one hour at room temperature. Finally, cells were washed in 1xPBS three times, washed briefly in DAPI and in sterile water then mounted onto slides with DAKO Fluorescent Mounting Medium (DAKO).

Mammospheres were fixed in ice-cold methanol and acetone (−20°C; 1∶1) for five minutes on ice [7]. Subsequently, the mammospheres were permeabilized and blocked in 0.1% Triton and 1% BSA in 1xPBS for 1 hour at RT. They were then washed in 1xPBS for 5 minutes three times. Primary antibodies against SPCA2, Orai1 and E-cadherin were diluted in 0.2% BSA in 1xPBS. Mammospheres were allowed to incubate in primary antibodies at 1∶250 dilutions overnight at 4°C. The next day, the mammospheres were washed gently in 1xPBS three times for 5 minutes at RT. Next, rabbit and mouse conjugated AlexaFluor secondaries (AlexaFluor 288 and 388, respectively) were incubated at a 1∶1000 dilution at RT for 1 hour in the dark, with slow agitation. Cells were washed with 1x PBS three times for 10 minutes with slow agitation in the dark for 5 minutes per wash. Cells were DAPI stained by brief submersion, washed in distilled, deionized water for 1 minute then the coverslips were permanently mounted to slides with fluorescent mounting medium (DAKO) and allowed to dry overnight in the dark.

### Lentiviral Production and Transfection

pLK0.1 plasmids were obtained from Sigma (Mission shRNA constructs) for SPCA2 and Orai1. HEK293T cells were inoculated with 1 µg plasmid DNA for each accessory packaging protein for lentiviral packaging. Media was removed from the cells after 72 hours and virus was purified using Lenti-X (Sigma) per the manufacturer’s instructions. Approximately 10 µg of virus was added to each well of a 6 well plate containing SCp2 cells at 50–70% confluence and allowed to incubate for 48 hours. The virus was then removed and cells were washed then inoculated with new media containing 500 µg/ml of puromycin for selection over 3 days. Once the cells were selected, they were grown in the selection media for two weeks then discarded. Each experiment was done within 5 passages of the cells to ensure complete RNA inhibition.

### Calcium Imaging

Cells were cultured as a monolayers or mammospheres on 25 mm circular coverslips. Briefly, cells were washed in sterile HBBS (Invitrogen) for five minutes at a time, three times. After washing, FURA2-AM (Invitrogen) was added at a final concentration of 1 µg/ml in imaging buffer (20 mM Hepes, 126 mM NaCl, 4.5 mM KCl, 2 mM MgCl_2_, 10 mM glucose at pH 7.4) containing 2 mM CaCl_2_. After incubation at room temperature for 20 minutes, cells were washed briefly in imaging buffer without calcium and 0.15% EGTA. Cells were then washed twice in imaging buffer without calcium to wash away residual EGTA and Ca^2+^. For mammosphere imaging, protocols were kept the same, however, after FURA2 loading, two 2 mM Ca^2+^ washes were added with slow agitation to remove FURA2 from the Matrigel, prior to washing in imaging buffer without calcium. Store dependent (SOCE) and independent (SICE) Ca^2+^ entry was measured as described [5]. Cells were excited at 340 nm and 380 nm, and Fura emission was monitored at 505 nm. For SOCE, cells were switched from 2 mM Ca^2+^ to nominally Ca^2+^ free recording buffer. Thapsigargin (2 µm) or ionomycin (2 µm) was added where indicated, and followed by readdition of 2 mM Ca^2+^ to measure store-dependent Ca^2+^ influx. For SICE, fluorescence was recorded from cells placed in nominally Ca^2+^ free recording buffer, followed by addition 2 mM Ca^2+^, activating store-independent Ca^2+^ influx.

## Supporting Information

Figure S1 **Control experiments for immunostaining of mammary gland.** Sections of lactating mouse mammary tissue were treated with a mixture of anti-SPCA1 or anti-SPCA2 antibody either with or without preincubated with the immunogenic peptide as indicated. Secondary antibody controls used in the absence of SPCA antibodies resulted in no specific signal, as shown. Nuclei are detected by DAPI staining as described in Methods.
(TIF)

Figure S2 **Morphology and Growth of SCp2 cells after transfection with shRNA constructs.** A. Growth of SCp2 cells after transfection with lentivirus carrying empty vector or shRNA against SPCA2 or Orai1 was monitored using MTT assay as described in Methods. No significant differences were observed following knockdown. B. Morphology of SCp2 cells following knockdown of SPCA2 and Orai1 is similar to that of control cells.
(TIF)

Figure S3 **Confocal sections of Immunofluorescence staining in Orai1 (A) and SPCA2 (B, C) knock down SCp2 cells.** A. Consecutive optical sections, starting from the bottom, of SCp2 cells knocked down for Orai channels stained with E-cadherin (red), SPCA2 (green) and DAPI (blue). Note the formation of tight junctions indicated by E-cadherin stain and normal shape of the cells. B. Consecutive optical sections, starting from the bottom, of SCp2 cells knocked down for SPCA2 stained with Orai1 (green) and DAPI (blue). Note the separation of Orai1 stain from the nucleus at the bottom and top sections. C. Confluent SCp2 cells treated with shEV (top) or shSPCA2 (bottom) stained for Orai1 (green) and DAPI (blue). Note the change in Orai1 localization from the cell boundaries (top) to circumnuclear (bottom).
(TIF)

Figure S4 **Estimation of Stored Ca^2+^ in SCp2 cells.** A. SCp2 cells were transferred to nominally Ca^2+^ free medium at time 0 and thapsigargin was added between 5–30 seconds as indicated. Ca^2+^ release was monitored by Fura2 (340/380 ratio). B. SCp2 cells were transferred to nominally Ca^2+^ free medium or not, at time 0 as indicated. Baseline Ca^2+^ before addition of thapsigargin was unchanged (*inset i*) Thapsigargin was added and Ca^2+^ release was monitored as fold-change relative to starting 340/380 ratios. The difference between the traces, indicated by gray shading, was plotted in *inset ii*. This indicates maximal SOCE resulting from thapsigargin mediated store release. Note that it is smaller than Ca^2+^ influx observed in Figure 6B.
(TIF)

Movie S1 **Consecutive confocal sections of a mammosphere immunostained with antibody against E-cadherin (red) and SPCA2 (green), with DAPI stain of nuclei (blue).**
(GIF)

Movie S2 **Consecutive confocal sections of a mammosphere immunostained with antibody against Orai1 (red) and SPCA2 (green), with DAPI stain of nuclei (blue).**
(GIF)

Movie S3 **Consecutive confocal sections of a mammosphere immunostained with antibody against Orai1 (red) and SPCA2 (green), with DAPI stain of nuclei (blue).**
(GIF)
