# Supplementary figures and images for: SPCA2 Regulates Orai1 Trafficking and Store Independent Ca2+ Entry in a Model of Lactation
Source: PLoS One. 2013 Jun 28;8(6):e67348. doi: 10.1371/journal.pone.0067348 (PMC3696057; doi:10.1371/journal.pone.0067348)

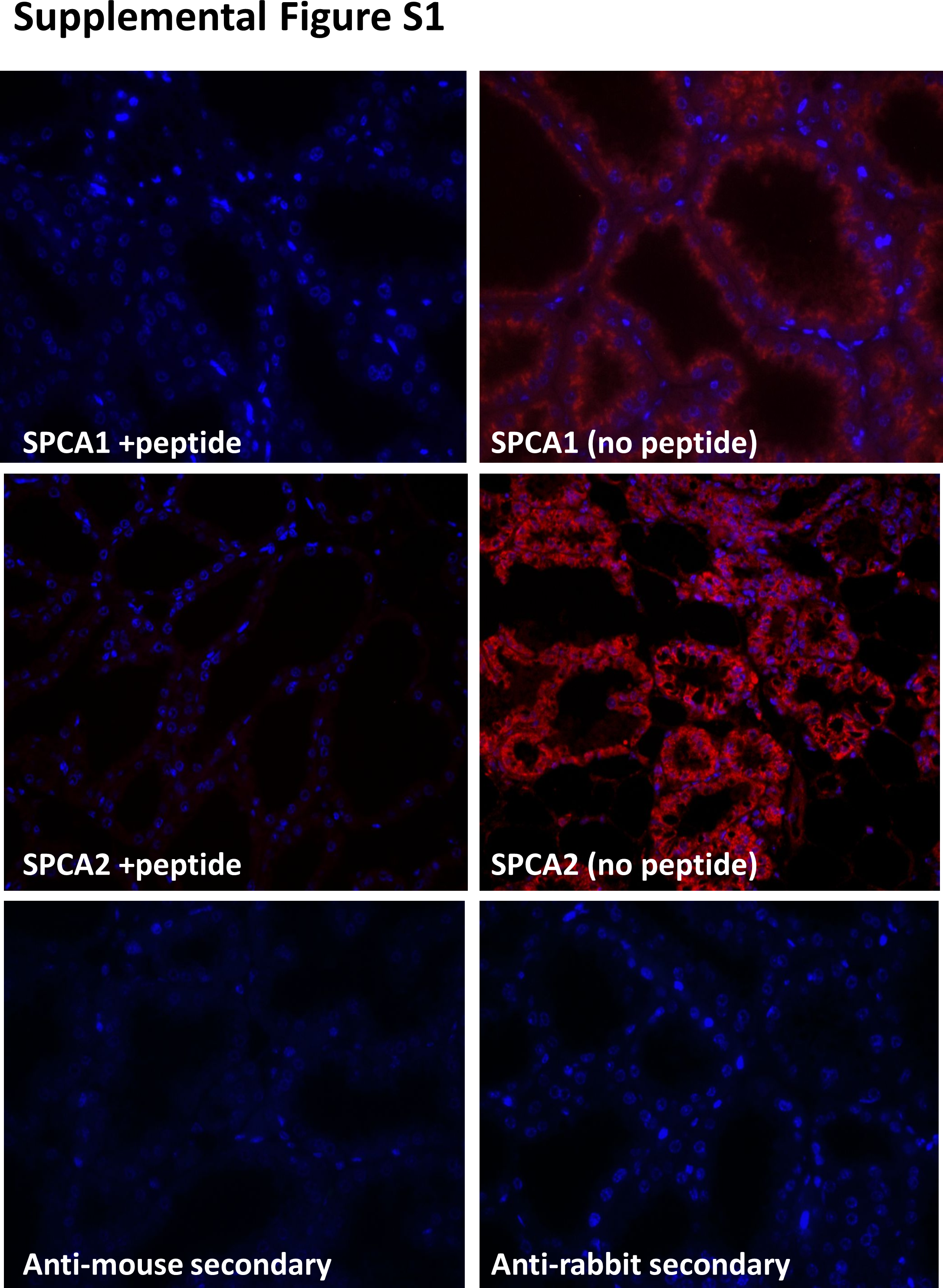

Supplement: Figure S1 — Control experiments for immunostaining of mammary gland. Sections of lactating mouse mammary tissue were treated with a mixture of anti-SPCA1 or anti-SPCA2 antibody either with or without preincubated with the immunogenic peptide as indicated. Secondary antibody controls used in the absence of SPCA antibodies resulted in no specific signal, as shown. Nuclei are detected by DAPI staining as described in Methods. (TIF) [file pone.0067348.s001.tif]

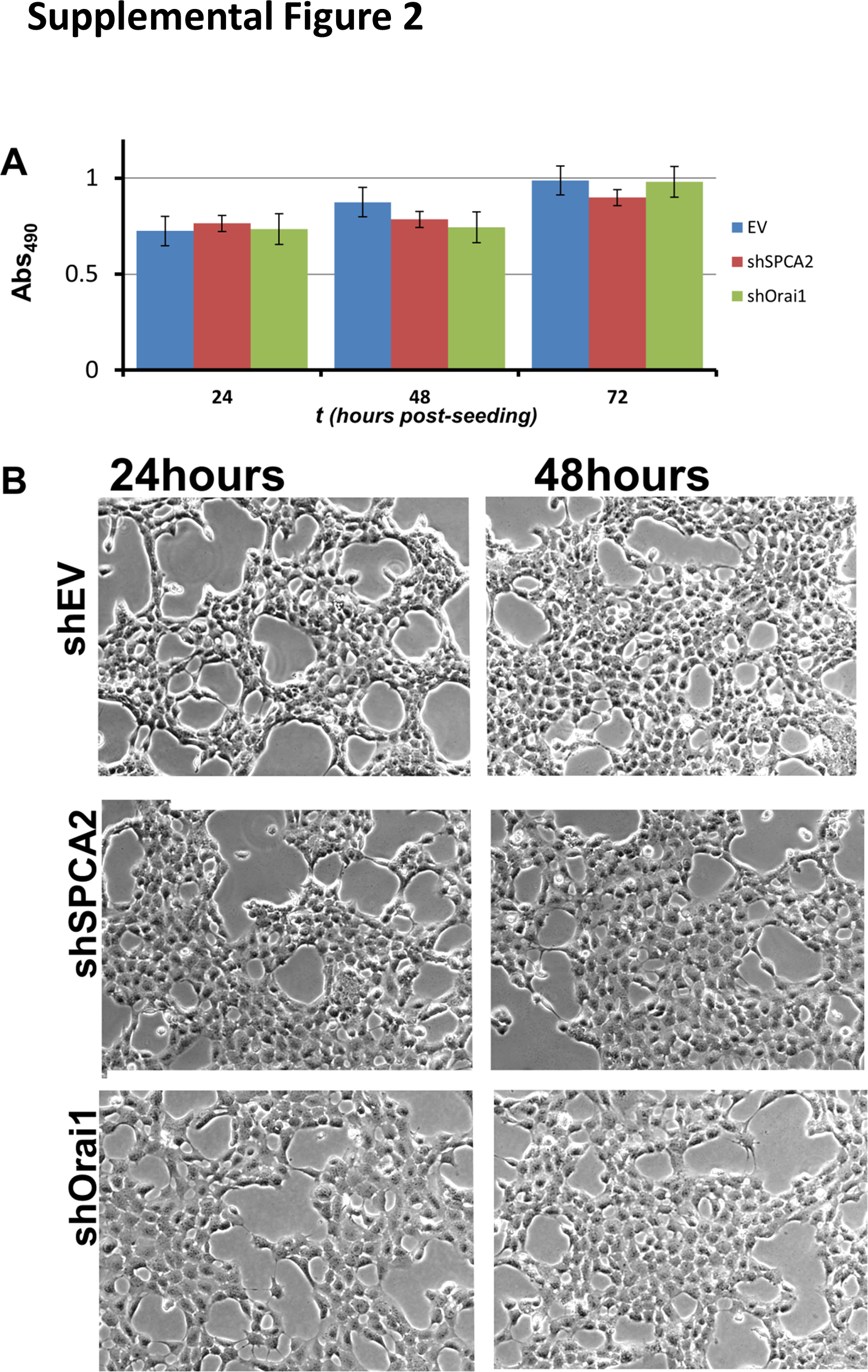

Supplement: Figure S2 — Morphology and Growth of SCp2 cells after transfection with shRNA constructs. A. Growth of SCp2 cells after transfection with lentivirus carrying empty vector or shRNA against SPCA2 or Orai1 was monitored using MTT assay as described in Methods. No significant differences were observed following knockdown. B. Morphology of SCp2 cells following knockdown of SPCA2 and Orai1 is similar to that of control cells. (TIF) [file pone.0067348.s002.tif]

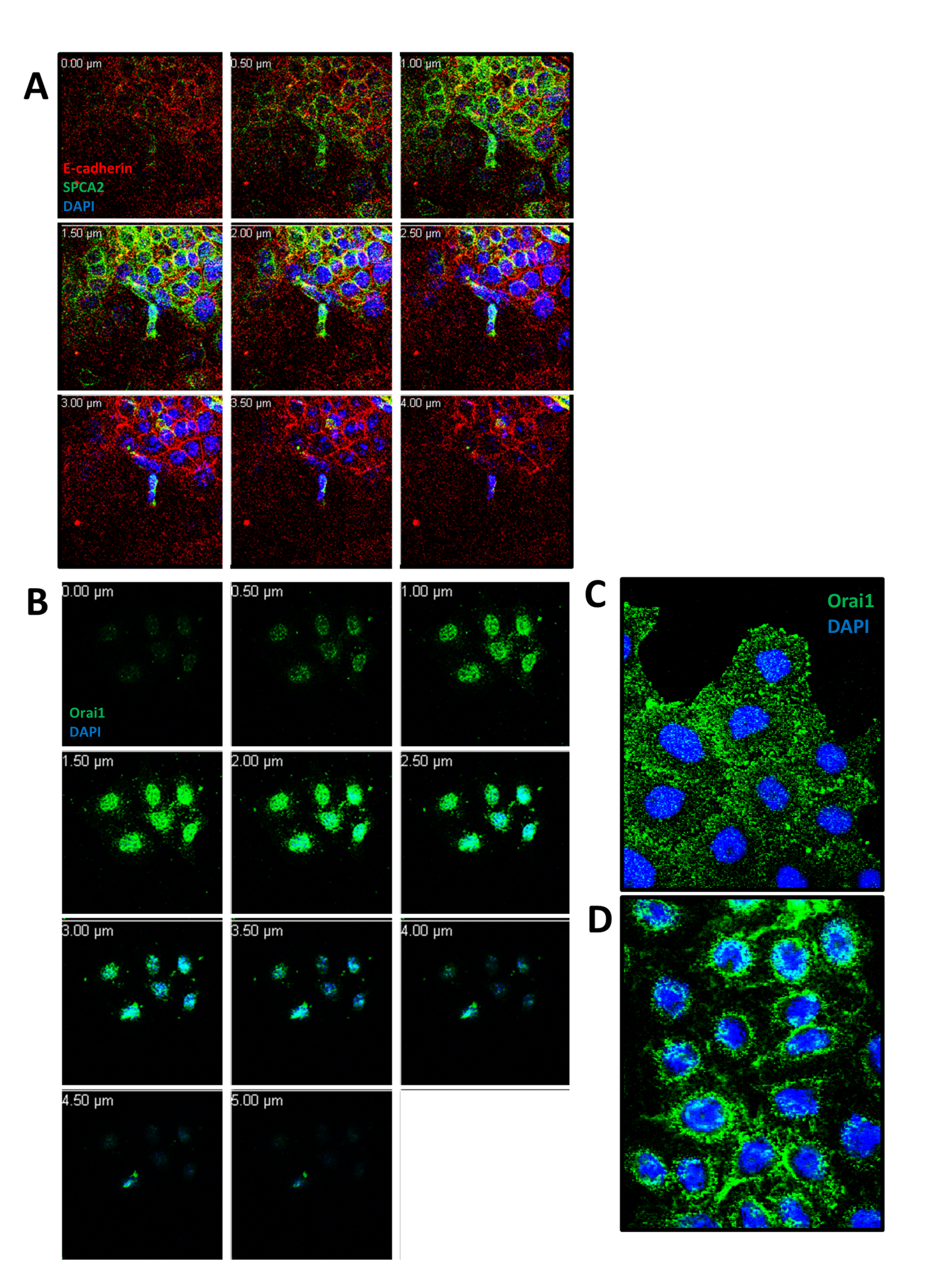

Supplement: Figure S3 — Confocal sections of Immunofluorescence staining in Orai1 (A) and SPCA2 (B, C) knock down SCp2 cells. A. Consecutive optical sections, starting from the bottom, of SCp2 cells knocked down for Orai channels stained with E-cadherin (red), SPCA2 (green) and DAPI (blue). Note the formation of tight junctions indicated by E-cadherin stain and normal shape of the cells. B. Consecutive optical sections, starting from the bottom, of SCp2 cells knocked down for SPCA2 stained with Orai1 (green) and DAPI (blue). Note the separation of Orai1 stain from the nucleus at the bottom and top sections. C. Confluent SCp2 cells treated with shEV (top) or shSPCA2 (bottom) stained for Orai1 (green) and DAPI (blue). Note the change in Orai1 localization from the cell boundaries (top) to circumnuclear (bottom). (TIF) [file pone.0067348.s003.tif]

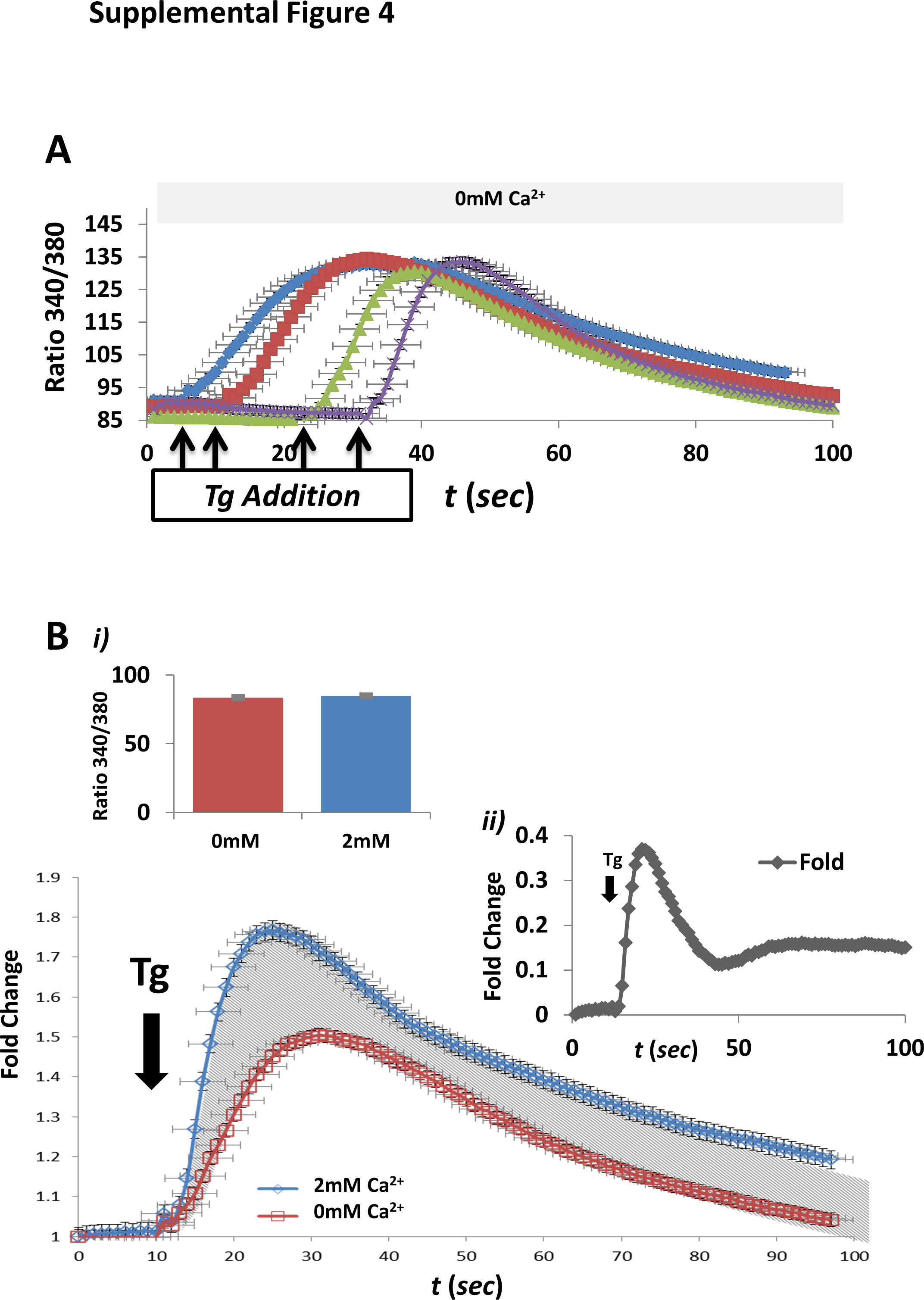

Supplement: Figure S4 — Estimation of Stored Ca2+ in SCp2 cells. A. SCp2 cells were transferred to nominally Ca2+ free medium at time 0 and thapsigargin was added between 5–30 seconds as indicated. Ca2+ release was monitored by Fura2 (340/380 ratio). B. SCp2 cells were transferred to nominally Ca2+ free medium or not, at time 0 as indicated. Baseline Ca2+ before addition of thapsigargin was unchanged (inset i) Thapsigargin was added and Ca2+ release was monitored as fold-change relative to starting 340/380 ratios. The difference between the traces, indicated by gray shading, was plotted in inset ii. This indicates maximal SOCE resulting from thapsigargin mediated store release. Note that it is smaller than Ca2+ influx observed in Figure 6B. (TIF) [file pone.0067348.s004.tif]

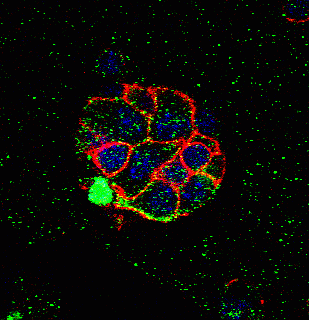

Supplement: Movie S1 — Consecutive confocal sections of a mammosphere immunostained with antibody against E-cadherin (red) and SPCA2 (green), with DAPI stain of nuclei (blue). (GIF) [file pone.0067348.s005.gif]

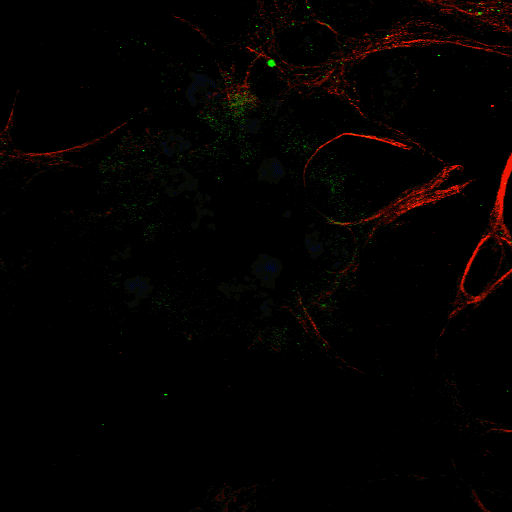

Supplement: Movie S2 — Consecutive confocal sections of a mammosphere immunostained with antibody against Orai1 (red) and SPCA2 (green), with DAPI stain of nuclei (blue). (GIF) [file pone.0067348.s006.gif]

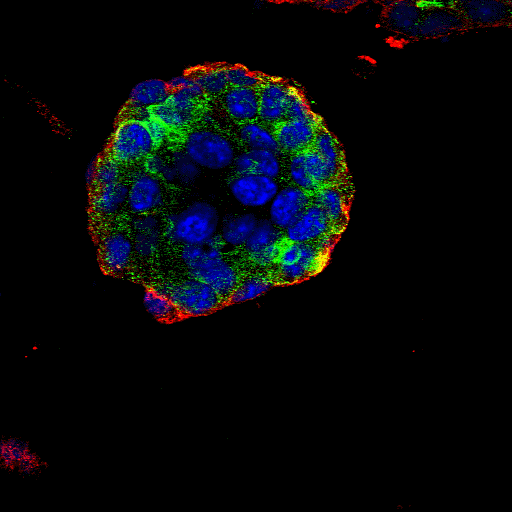

Supplement: Movie S3 — Consecutive confocal sections of a mammosphere immunostained with antibody against Orai1 (red) and SPCA2 (green), with DAPI stain of nuclei (blue). (GIF) [file pone.0067348.s007.gif]
